# Feasibility and suitability of a graded exercise test in patients with aggressive hemato-oncological disease

**DOI:** 10.1007/s00520-021-06035-w

**Published:** 2021-02-05

**Authors:** Anja Großek, Thomas Elter, Max Oberste, Florian Wolf, Niklas Joisten, Philipp Hartig, David Walzik, Friederike Rosenberger, David Kiesl, Patrick Wahl, Wilhelm Bloch, Philipp Zimmer

**Affiliations:** 1 Department of Molecular and Cellular Sports Medicine, Institute of Cardiovascular Research and Sports Medicine, German Sport University Cologne, Cologne, Germany; 2 Department I of Internal Medicine, Center for Integrated Oncology Köln Bonn, University of Cologne, Cologne, Germany; 3 Division for Performance and Health (Sport Medicine), Department of Sport and Sport Science, Technical University Dortmund, Otto-Hahn-Str. 3, 44227 Dortmund, Germany; 4 Working Group Exercise Oncology, Department of Medical Oncology, National Center for Tumor Diseases (NCT), Heidelberg University Hospital, Heidelberg, Germany; 5 University Clinic for Hematology and Internal Oncology, Kepler University Hospital, Linz, Austria

**Keywords:** Acute leukemia, Lymphoma, Exercise test, ACSM criteria

## Abstract

**Purpose:**

Physical activity promises to reduce disease-related symptoms and therapy-related side effects in patients suffering from aggressive lymphoma (L) or acute leukemia (AL). For an efficient training program, determination of patients’ physical capacity with a purposive exercise test is crucial. Here, we evaluated the feasibility and suitability of a graded exercise test (GXT) frequently applied in patients suffering from solid tumors by assessing whether patients achieved criteria for maximal exercise testing according to the American College of Sports Medicine (ACSM).

**Methods:**

The GXT was performed by 51 patients with an aggressive L or AL prior to the start or in the earliest possible phase of high-dose chemotherapy, following a recommended protocol for cancer patients, starting at 20 Watts (W), with an increase of 10 W/min until volitional exhaustion. Subsequently, we investigated whether the following ACSM criteria were fulfilled: (1) failure of heart rate to increase despite increasing workload, (2) post-exercise capillary lactate concentration ≥ 8.0 mmol L^−1^, (3) rating of perceived exertion at exercise cessation > 17 on the 6–20 Borg Scale.

**Results:**

Out of 51 patients, two, six, and 35 participants met the first, second, and third criterion, respectively. No relevant relationships between the completion of the criteria and patients’ characteristics (e.g., gender, age) were found.

**Conclusion:**

Although results of this study suggest a general feasibility of the applied GXT, the ACSM criteria were not met by the majority of the participants. Therefore, this study raises doubts about the suitability of the GXT protocol and the ACSM criteria for this group of patients.

## Introduction

Hemato-oncological diseases such as acute leukemia (AL including acute lymphocytic leukemia (ALL) and acute myeloid leukemia (AML)) or aggressive lymphoma (L) have a high impact on a patient’s life, both physically and mentally. Despite therapeutic improvements, only about 30% of the AL patients survive the first 10 years after diagnosis [1]. The dramatically fast progressing disease demands a rapid hospitalization for initiation of intensive induction chemotherapy, which usually lasts 4 to 8 weeks. Besides symptoms caused by the disease itself, such as anemia, fever, or vulnerability to infections, patients have to deal with treatment-related side effects such as depression or fatigue [2]. The combination of disease, hospitalization, and therapy side effects leads to a decrease in physical activity, an increase of psychological stress, and an overall reduction in health-related quality of life [3–5]. Currently, research data suggests that physical activity is the most promising counter to alleviate these side effects and it has already been nominated as medicine to prevent cancer and support cancer therapy [6]. Nevertheless, the “dose” of physical activity has to be set individually in dependence of the patients’ type of disease, therapy, needs, and physical capacity [7, 8]. According to the FITT criteria, this “dose” consists of frequency, intensity, time, and type of physical activity and, as in medication, it is important to determine and adjust these FITT criteria to receive optimal outcome [9]. As these parameters are very sensitive to individual physical capacity, accurate measurements of the patients’ status are necessary to set an individually appropriate dose. Endurance and resistance training are the two major types of physical activity, which have been tested on safety, feasibility, and efficiency in oncological patients [6, 8]. With regard to aggressive hemato-oncological diseases, the scientific focus has been set on mixed modality exercise (mainly including resistance and aerobic exercise) as well as endurance training only [10–12]. To set the right training intensity in endurance training, the physical capacity of the patients has to be measured. In this context, cardiopulmonary exercise testing (CPET) is considered gold standard. Nevertheless, this exercise test not only requires above-standard equipped hospitals and specifically trained personal infrastructure, but also adds additional stress to this vulnerable group of patients. To avoid any further burden for patients and because CPETs are often not comprehensively available, a graded exercise test (GXT) is commonly used to investigate the physical capacity of the patients. The safety and feasibility of the GXT with and without gas analysis have been demonstrated in patients with various cancer types such as breast or prostate cancer [13]. Furthermore, different protocols have also been tested in hemato-oncological patients throughout different stages of their therapy [14, 15]. To standardize the assessment methods of exercise in cancer patients, Scharhag-Rosenberger et al. [13] set up recommendations for a GXT protocol amongst others.

The aim of this investigation is (1) to test the safety and feasibility of the recommended GXT protocol in patients with an aggressive hemato-oncological disease prior to the start of the induction/high-dose chemotherapy; and (2) evaluate whether the participants reached their maximal physical capacity during the recommended protocol based on the American College of Sports Medicine (ACSM) criteria for maximal exercise tests [16].

## Methods

This cross-sectional investigation is part of a larger randomized controlled trial [17], which was carried out between May 2016 and February 2019 at the University Hospital Cologne, Germany. After hospitalization to the Clinic for Hematology and Oncology, patients were screened and, if eligible, informed about the study. If the patient stated interest, the patient and the attending doctor were asked to sign the written informed consent form to participate in the trial. The date for the GXT was scheduled immediately, taking into consideration the priority of hospital examinations and the protocol of the larger trial [17] as well as the health status of the participants, which needed to allow for high-intensity exercise. The time point was chosen based on the protocol of the larger trial. Additionally, this time point has the most value for sport- and physiotherapist as they normally start their therapy as early as possible. Ethical approval for the trial was obtained from the Ethics Commission of the University Hospital Cologne, Germany (15-161); the study was registered at the WHO trial register on 26.01.2016 (DRKS00007824) and performed in accordance with the latest Declaration of Helsinki.

### Participants

Patients were eligible for the study if they were diagnosed with AL or aggressive L and planned for induction/high-dose chemotherapy (such as B-ALL-protocol, GMALL-protocol) or a chemotherapy regime following a stem cell transplantation (such as DHAP-induction, 7+3, FLAG, S-HAM). The exclusion criteria were age under 18, comorbidities that contraindicated for exercise testing, such as coronary artery disease, heart failure (NYHA >3), metastases in the central nervous system, and neurodegenerative diseases.

### ACSM criteria

The ACSM criteria for a maximal exercise test serve as a guideline to verify the research question [16]. These criteria are (1) a plateau in oxygen uptake (V̇O_2_) with increased workload, (2) failure of heart rate (HR) to increase with increases in workload (increase of 0 beats between the second last and the last minute), (3) a post-exercise capillary lactate concentration (bLA) ≥ 8.0 mmol L^−1^, (4) a rating of perceived exertion at peak exercise > 17 on the 6–20 Borg Scale, and (5) a peak respiratory exchange ratio (RER_peak_) ≥ 1.10. Due to practical reasons and to reduce the stress level of the participants, no respiratory gas analysis was conducted wherefore criteria (1) and (5) were excluded from further analysis. Although the ACSM does not report a number of criteria which have to be accomplished for a successful testing, previous literature suggests that the criteria chosen for the analysis should not be investigated on their own but interpreted in the context of the remaining criteria if possible [18, 19]. Therefore, the analysis not only focused on differences in participants that fulfilled one criterion against those who did not fulfil the same criterion but participants were also further grouped into those who at least fulfilled two of the remaining three possible criteria against those who only fulfilled one or none out of three.

Besides the ACSM criteria, further criteria to estimate whether maximal intensity was reached are used and discussed in the literature [14, 20–24]. Therefore, the analysis was extended by (a) measuring bLA 3 min after completion of the test, (b) examining HR changes between the last two time points of measurement of less than 2%, (c) investigating whether maximal HR reached the calculated maximal HR of 220 minus the patient’s age [25, 26], as well as (d) the calculated maximal HR with the equation 200 minus the patient’s age [23].

### Assessments

Prior to the GXT, the current health status was checked with the patient and the attending doctor. The patient was not eligible to participate in the test if any of the following occurred: (1) blood platelet count below 10,000*μL^−1^, (2) hemoglobin values below 7 g*dL^−1^, (3) fever, infections or diarrhea, or (4) the patient did not agree to participate or felt unwell. The test would then be rescheduled if possible. The GXT was performed on a bicycle ergometer (Basic Clubbike Sit, Milon Industries GmbH Miltronic, Emersacker, Germany) in accordance with recommended guidelines for oncologic patients [13]. As suggested in these guidelines, the test started at 20 Watts (W), with an increase of 10 W * min^−1^ until volitional exhaustion. During the test, a pedal frequency of 60–70 revolutions per minute was kept following the guidelines and the manufacturer’s advice. The test was conducted by trained sport or medical students. HR and blood lactate from the hyperaemized earlobe were measured in rest before the test as well as at the time of exhaustion and 3 min after completing the test. Furthermore, at each stage of the protocol and at the end, HR and perceived exertion were monitored using a heart rate monitor (Polar FT1, Polar Electro Oy, Kempele, Finland), and the rate of perceived exertion Original Borg Scale [19] (RPE). The blood lactate concentration was analyzed at a laboratory of the German Sports University (Biosen S_line Lab+, EKF Diagnostic, Barleben, Germany).

Besides these test variables, socio-demographic data were collected using the medical record and an evaluation sheet. In addition to accounting for age, gender-related physical fitness level was calculated and categorized into normal, slightly reduced, reduced, strongly reduced, and greatly reduced, using the peak power reached during the GXT [23]. Furthermore, participants were asked to rank their self-perceived amount of physical activity and exercise level on a 10-cm visual analogue scale. This value was categorized as follows: 0 cm to 3.3 cm coded for inactive, 3.4 cm to 6.6 cm coded for active, and 6.7 cm to 10 cm coded for very active.

### Statistical analysis

The data analysis was performed using IBM SPSS version 26. Descriptive statistics and frequency tables were tabulated to describe participants’ characteristics. Results display cross-tabulations between the ACSM criteria and socio-demographic variables. Fisher’s exact test for small sample sizes was performed to exactly examine relationships between variables. Additionally, Cramer’s *V* was conducted to express the strength of associations between the dependent and nominally scaled independent variables.

## Results

The GXT was carried out by 51 participants of the 72 participants from the larger trial. Of the missing 21 participants, 19 did not attend the GXT (due to drop out, physical and/or psychological stress besides others) and three stopped the test (due to pain in the knee, nausea, and dizziness) before reaching subjective exhaustion. No adverse events occurred. Table 1 shows the participants’ characteristics and the GXT results. The majority of the participants were diagnosed with AML (70.6%), followed by ALL (15.7%) and L (13.7%). Slightly more men (*n*=27) than women (*n*=24) participated in this investigation. The hemoglobin averaged out at 9 g*dL^−1^ (± 2.17) and the physical fitness of 9 participants compared with the age-related controls was normal. Blood lactate concentration at exhaustion could not be determined in 13 participants and blood lactate concentration 3 min after the end of the testing could not be determined in 10 participants due to error within the blood drawing (*n*=8) and refusal of blood drawing (*n*=2). Tables 2 and 3 present the characteristics of the participants defined by the accomplishment of the ACSM criteria and the extended criteria, respectively.

**Table 1:** Patient characteristics

	Mean ± SD (range)	*N* (%)
Descriptive data		
Age (years)	47.5 ± 15.8 (18–75)	
Height (cm)	174.8 ± 10.4 (148–196)	
Weight (kg)	81.5 ± 15.9 (52–124)	
BMI (kg*m^−2^)	26.5 ± 4.0 (19.2–39.8)	
Hemoglobin (g*dL^−1^)	9 ± 2.13 (7.0–14.5)	
Education (years)	11.6 ± 1.7 (8–13)	
Sex		
Male		27 (52.9)
Female		24 (47.1)
Smoker		
Yes		12 (27.9)
No		31 (72.1)
Clinical data		
Diagnosis		
AML		36 (70.6)
ALL		8 (15.7)
Lymphoma		7 (13.7)
Type of therapy		
S-HAM		30 (58.8)
GMALL		11 (21.6)
Other		10 (19.6)
Performance data		
HR_rest_ (bpm)	89 ± 19.2 (59–130)	
Blood lactate_rest_ (mmol*L^−1^)	1.85 ± 0.82 (0.7–3.88)	
HR_max_	145.39 ± 21.03 (88–182)	
Blood lactate_max_ (mmol*L^−1^)	5.71 ± 2.91 (0.85–13.34)	
*P*_max_ (W)	95.88 ± 34.42 (30–160)	
Physical fitness		
Normal		9 (17.6)
Slightly reduced		4 (7.8)
Reduced		23 (45.1)
Strongly reduced		12 (23.5)
Greatly reduced		3 (5.9)

*SD* standard deviation, *BMI* body mass index, *AML* acute myeloid leukemia, *ALL* acute lymphoblastic leukemia, *S-HAM* sequential high-dose cytarabine and mitoxantrone, *GMALL* German multicenter acute lymphoblastic leukemia, *HR* heart rate, *P*_*max*_ maximal power output

**Table 2:** Cross table of ACSM criteria and participant characteristics

	Failure of HR to increase		Lactate concentration ≥ 8 mmol L^−1^		RPE > 17		2 out of 3 ACSM criteria	
	Yes, *n*	No, *n*	Yes, *n*	No, *n*	Yes, *n*	No, *n*	Yes, *n*	No, *n*
Overall	2	49	6	32	35	16	7	44
Gender								
Male	2	25	3	18	19	8	5	22
Female	0	24	3	14	16	8	2	22
Cramer’s *V* (Fisher’s exact *p*)	.190 (.492)		.046 (1.00)		.040 (1.00)		.148 (.425)	
Age group								
≤ 30	1	8	0	7	8	1	1	8
31–45	0	12	1	6	9	3	1	11
46–60	1	18	4	14	14	5	5	14
> 60	0	11	1	5	4	7	0	11
Fisher’s exact *p*	.657		.761		.076		.236	
Diagnosis								
AML	0	36	5	21	26	10	4	32
ALL	0	8	0	6	3	5	0	8
Lymphoma	2	5	1	5	6	1	3	4
Cramer’s *V* (Fisher’s exact *p*)	.507 (.016)		.189 (.805)		.306 (.091)		.357 (.042)	
Type of therapy								
S-HAM	0	30	4	17	21	9	3	27
GMALL	2	9	0	9	7	4	2	9
Other	0	10	2	6	7	3	2	8
Cramer’s *V* (Fisher’s exact *p*)	.385 (.078)		.250 (.429)		.056 (.917)		.131 (.617)	
Smoker								
Yes	0	12	1	9	11	1	1	11
No	1	30	4	18	17	14	4	27
Cramer’s *V* (Fisher’s exact *p*)	.096 (1.00)		.104 (.656)		.347 (.033)		.064 (1.00)	
BMI								
Normal weight	0	17	2	8	9	8	2	15
Overweight	2	23	2	19	19	6	3	22
Obese class I	0	8	2	5	6	2	2	6
Obese class II	0	1	0	0	1	0	0	1
Fisher’s exact *p*	.667		.424		.372		.773	
Comorbidities								
0	0	22	5	11	17	5	5	17
1–2	2	19	1	15	15	6	2	19
>2	0	8	0	6	3	5	0	8
Fisher’s exact *p*	.456		.134		.141		.341	
Physical activity								
Inactive	1	2	0	3	2	1	1	2
Active	1	15	1	9	9	7	2	14
Very active	0	26	4	16	19	7	3	23
Fisher’s exact *p*	.052		.784		.549		.472	
Exercise								
Inactive	2	9	1	7	8	3	3	8
Active	0	16	2	10	10	6	2	14
Very active	0	18	2	11	12	6	1	17
Fisher’s exact *p*	.056		1.00		.922		.211	

*AML* acute myeloid leukemia, *ALL* acute lymphoblastic leukemia, *S-HAM* sequential high-dose cytarabine and mitoxantrone, *GMALL* German multicenter acute lymphoblastic leukemia, *BMI* body mass index, *HR* heart rate, *RPE* rate of perceived exertion. The self-perceived amount of physical activity and exercise was rated on a 10-cm visual analogue scale and then categorized into “inactive” (0–3.3 cm), “active” (3.4–6.6 cm), and “very active” (6.7–10 cm)

**Table 3:** Cross table of extended ACSM criteria and participant characteristics

	Lactate concentration 3 min after GXT ≥ 8 mmol L^−1^		HR changes in the last minute < 2%		Reached HR_max_ with 220−age		Reached HR_max_ with 200−age	
	Yes, *n*	No, *n*	Yes, *n*	No, *n*	Yes, *n*	No, *n*	Yes, *n*	No, *n*
Overall	9	32	10	41	1	49	22	28
Gender								
Male	6	15	7	21	1	25	12	14
Female	3	17	3	21	0	24	10	14
Cramer’s *V* (Fisher’s exact *p*)	.164 (.454)		.169 (.300)		.137 (.100)		.045 (.783)	
Age group								
≤ 30	2	5	2	7	0	9	2	7
31–45	1	7	2	10	0	12	6	6
46–60	5	13	4	15	1	18	10	9
> 60	1	7	2	9	0	10	4	6
Fisher’s exact *p*	.755		1.000		1.000		.495	
Diagnosis								
AML	7	22	7	29	0	35	14	21
ALL	1	5	2	6	0	8	4	4
Lymphoma	1	5	1	6	1	6	4	3
Cramer’s *V* (Fisher’s exact *p*)	.082 (1.00)		.073 (1.00)		.354 (.140)		.129 (.749)	
Type of therapy								
S-HAM	6	18	7	23	0	29	12	17
GMALL	1	8	2	9	1	10	5	6
Other	2	6	1	9	0	10	5	5
Cramer’s *V* (Fisher’s exact *p*)	.139 (.767)		.130 (.802)		.269 (.420)		.069 (.926)	
Smoker								
Yes	2	10	2	10	1	11	5	7
No	5	17	6	25	0	30	13	17
Cramer’s *V* (Fisher’s exact *p*)	.072 (1.00)		.031 (1.00)		.247 (.286)		.015 (.921)	
BMI								
Normal weight	3	8	2	15	0	16	9	8
Overweight	4	18	6	19	1	23	9	15
Obese class I	2	5	2	6	0	8	4	4
Obese class II	0	1	0	1	0	1	0	1
Fisher’s exact *p*	.744		.700		1.00		.705	
Comorbidities								
0	6	11	4	18	0	21	10	11
1–2	2	16	4	17	0	21	8	13
>2	1	5	2	6	1	7	4	4
Fisher’s exact *p*	.235		.901		.160		.801	
Physical activity								
Inactive	0	3	1	2	0	3	2	1
Active	3	9	3	13	0	15	6	9
Very active	5	15	5	21	0	26	12	14
Fisher’s exact *p*	1.00		.719		-		.636	
Exercise								
Inactive	1	8	2	9	0	10	4	6
Active	3	10	2	14	0	16	8	8
Very active	4	9	5	13	0	18	8	10
Fisher’s exact *p*	.545		.579		-		.928	

*AML* acute myeloid leukemia, *ALL* acute lymphoblastic leukemia, *S-HAM* sequential high-dose cytarabine and mitoxantrone, *GMALL* German multicenter acute lymphoblastic leukemia, *BMI* body mass index, *HR* heart rate. The self-perceived amount of physical activity and exercise was rated on a 10-cm visual analogue scale and then categorized into “inactive” (0–3.3 cm), “active” (3.4–6.6 cm), and “very active” (6.7–10 cm)

### Heart rate

Of the 51 participants, two (3.9%) acquired the state where the HR did not increase any further in the test. Both participants were male and diagnosed with L. The Fisher’s exact test between failure of further increase in HR and gender did not reach statistical significance. A significant test result for a medium strong association was found for the failure of HR increase and the diagnosis (Cramer’s *V* = 0.507, Fisher’s exact test value = 7.417, *p*=0.016). Furthermore, the associations between the amount of physical activity, the level of exercise, and the failure of the HR to further increase almost reached the 5% significance level using the Fisher’s exact test (Fisher’s exact test value = 5.373, *p*=0.052; Fisher’s exact test value = 4.169, *p*=0.056, respectively). No significant results were found taking the enlarged criteria (a), (b), (c), and (d) into account.

### Lactate concentration

The bLA was successfully measured in 38 participants. Six (15.8%) participants revealed a bLA of ≥ 8 mmol*L^−1^ after completing the GXT. Neither significant results between the bLA measured directly after exertion nor between bLA measured 3 min after exhaustion ≥ 8 mmol*L^−1^ and any of the independent variables were found.

### Rating of perceived exertion

The perceived exertion of 35 participants (68.8%) exceeded a rank of 17 on the Borg Scale. Tests showed a low to medium significant association between whether participants smoked and reporting a Borg Scale value > 17 (Cramer’s *V* = 0.347, Fisher’s exact test value = 5.166, *p*=0.033). Participants, who aborted the GXT without reporting a Borg Scale > 17, stated the following reasons to end the test: legs feeling queasy, difficulties to breathe, nausea, knee pain, and insecurity at a hearth rate over 140.

### Accomplishment of two out of three possible ACSM criteria

After participants were grouped into those who fulfilled at least two of the three criteria (*n*=7) against those who fulfilled only one or none (*n*=44; *n*=15; and *n*=29, respectively), a low to medium significantly strong association between diagnosis and accomplishment of two out of three criteria was found (Cramer’s *V* = 0.357, Fisher’s exact test value = 4.971, *p*=0.042). No other significant associations were found for the remaining independent variables (gender, age, type of therapy, smoking behavior, BMI, comorbidities, physical activity, exercise) after the statistical analysis.

## Discussion

This is the first investigation testing the ACSM criteria on the established and recommended GXT protocol for individuals with cancer in patients with aggressive hemato-oncological diseases prior to high-dose chemotherapy. Although the GXT was feasible and safe, the results indicated that the minority of participants achieved a maximal effort according to the criteria of the ACSM. Reasons why participants reached exhaustion could not clearly be attributed to any of the investigated variables as they predominantly did not reach statistical significance. The only significant results were found for associations between being a smoker and perceived exhaustion, with smokers reaching a higher rating of perceived exhausting more often than non-smokers and lymphoma patients, who fulfilled two out of three criteria, reaching a heart rate plateau more likely than leukemia patients.

Throughout the literature, different test procedures were used to measure the physical fitness level of patients with an aggressive hemato-oncological disease [4, 10, 27]. Some of these, such as the Short Physical Performance Battery [27], the 6-minute Walk Test [4], as well as the 12-minute Walking Distance Test [10], can only be used to give an indicator for the patients’ level of fitness or to detect intrapersonal changes in time, but they cannot be used to determine the training intensity. Trials, which did not bind testing results to the training intensity, frequently relied on a percentage of the heart rate reserve (HRR) to set the training intensity [4, 28, 29]. Story et al. [14] tested the HRR method against the HRs obtained during a CPET in adults with AL undergoing treatment and stated that the HRR method is not a valid method to regulate the training intensity. Other studies applied a modification of the GXT performed in this study and utilized the test results to set the exercise intensity [30, 31]. However, they used 180 minus the age of the participant as a cut-off value for the test, which might have led to an underestimation of the physical capacity of the participant.

Schneider et al. [22] conducted the same GXT protocol as used in our investigation (including CPET) in a very different patient group consisting of prostate and breast cancer survivors. Besides a verification test, they considered the test maximal when at least two out of the following four criteria occurred (1) RER_peak_ ≥ 1.1, HR_peak_ ≥ 200 minus age, peak bLA ≥ 8 mmol L^−1^, and RPE ≥ 18. Out of their 40 participants, only 18% failed to meet these criteria whereas in the present investigation, 68.6% of the participants failed to reach exhaustion according to the criteria mentioned above. This leads to the assumption that the stage of therapy alongside the kind of neoplasm plays a crucial role in successfully completing the recommended exercise test based on the exhaustion criteria.

Current literature not only states the safety and feasibility of the graded exercise test, as performed in this investigation, but also recommends this test for oncological patients [13]. The data gathered in this study supports the safety and feasibility of the GXT as no adverse events occurred and a large majority of participants finished the test according to the protocol. However, adverse events are rare in general and less likely observed in studies with a limited sample size. A larger sample size for future investigations is desirable. Nevertheless, the purpose of the conduction might be unrewarding as the majority of the participants do not fulfill the ACSM criteria for exhaustion. Thus, it is questionable whether or not the test results really state the maximal capacity of the participants and if these criteria are valid for this group of patients after all. Furthermore, the results predominantly suggest the independence of the criteria and the included independent variables, so that meaningful test results were not even found in subgroups where they were expected the most, i.e., for the younger and healthier patients. In summary, our findings raise the question whether participants reach their maximal physical capacity in the performed GXT protocol.

In case they did, other criteria than those of the ACSM are warranted. As they are systemic diseases, AL and aggressive L have a high impact on both single body parts and the whole organism, which might not only lead to a reduced maximal physical capacity but also to a shift in stress limits and therefore might imply the need of different testing criteria. Despite the results of the GXT and the informative value with regard to training, the “dose” of endurance exercise stays questionable.

In case they did not, another GXT protocol or other testing procedures might be more suitable. Besides a GXT, a steep ramp test (SRT) promises to be a valid and reliable test for determining the physical capacity in cancer patients [32]. Nevertheless, recent literature states that the prediction of the peak oxygen consumption and peak power output are adequate at the group level but not on individual level [33]. A combination of a SRT with an endurance test [34] might be an adequate solution to reach the maximal physical capacity of this group of patients, though it still has to be tested in future investigations.

Results of the current study should be read within the context of its limitations. First of all, only three out of the five ACSM criteria could be tested due to the lack of respiratory gas analysis. This decision was mainly taken to reduce participants’ stress as their physical and psychological condition is already weakened. Second, this investigation was part of a larger trial including an exercise intervention; a selection bias in exercise-interested participants cannot be ruled out. Third, the sample size is relatively small.

## Conclusion

Overall, it can be stated that a GXT with the recommended protocol is safe and feasible for patients suffering from an aggressive form of leukemia or lymphoma. However, only a small subsample was able to fulfill any of the ACSM criteria. Thus, this analysis raises the question of the expediency of either the GXT or the ACSM criteria in this group of patients. The explanatory power of the GXT results seems limited and the transfer of the test results to the training settings not advisable at the current state.

## Data Availability

Not applicable.
